# The Auditory Agnosias: a Short Review of Neurofunctional Evidence

**DOI:** 10.1007/s11910-023-01302-1

**Published:** 2023-09-25

**Authors:** Gabriele Miceli, Antea Caccia

**Affiliations:** 1 Professor of Neurology, Center for Mind/Brain Studies, University of Trento, Trento, Italy; 2 University of Milano – La Bicocca, Milan, Italy

**Keywords:** Pure auditory agnosia, Global auditory agnosia, Verbal auditory agnosia, Nonverbal auditory agnosia, Auditory pathways, Hemispheric asymmetries

## Abstract

**Purpose of Review:**

To investigate the neurofunctional correlates of pure auditory agnosia and its varieties (global, verbal, and nonverbal), based on 116 anatomoclinical reports published between 1893 and 2022, with emphasis on hemispheric lateralization, intrahemispheric lesion site, underlying cognitive impairments.

**Recent Findings:**

Pure auditory agnosia is rare, and observations accumulate slowly. Recent patient reports and neuroimaging studies on neurotypical subjects offer insights into the putative mechanisms underlying auditory agnosia, while challenging traditional accounts.

**Summary:**

Global auditory agnosia frequently results from bilateral temporal damage. Verbal auditory agnosia strictly correlates with language-dominant hemisphere lesions. Damage involves the auditory pathways, but the critical lesion site is unclear. Both the auditory cortex and associative areas are reasonable candidates, but cases resulting from brainstem damage are on record. The hemispheric correlates of nonverbal auditory input disorders are less clear. They correlate with unilateral damage to either hemisphere, but evidence is scarce. Based on published cases, pure auditory agnosias are neurologically and functionally heterogeneous. Phenotypes are influenced by co-occurring cognitive impairments. Future studies should start from these facts and integrate patient data and studies in neurotypical individuals.

**Supplementary Information:**

The online version contains supplementary material available at 10.1007/s11910-023-01302-1.

## Introduction

The term auditory agnosia denotes disorders of auditory input processing that follow damage to the central nervous system and cannot be explained by peripheral hearing loss. In extreme forms, these disorders result in cortical deafness—the patient fails to react to auditory stimuli or responds occasionally and often inappropriately. In more common, less severe forms auditory input processing disorders result in clinically distinguishable disorders, characterized by the disruption of all types of input (generalized auditory agnosia) or by disproportionate difficulty for speech (verbal auditory agnosia, also called word deafness) or environmental sounds (non-verbal auditory agnosia)^1^.

Auditory agnosia is relatively frequent in aphasic syndromes, in which it typically co-occurs with additional language comprehension and production impairments. In rare instances, however, it is observed in “pure” forms, in which difficulties processing auditory input dissociate from disorders of visual language processing and, occasionally, from speech production impairments. Studies of these disorders shed light on the neurofunctional mechanisms involved in the processing of auditory input. The present review focuses on acquired pure auditory agnosias in the adult. In the manuscript, the acronym AA denotes pure auditory input processing impairments, irrespective of whether generalized or selective, and the acronyms gAA, vAA, and nvAA refer to the generalized, verbal and non-verbal clinical varieties of AA, respectively.

Owing to the rarity of the disorder, reports of pure AA have accumulated almost invariably one case at a time. The present review is based on the 116 cases published between 1893 and December 2022 that provide behavioral and anatomical information (see Supplementary Materials). Papers that merely list damaged structures are considered when discussing gross anatomo-functional correlations, whereas studies that provide detailed neuroimaging or autopsy documentation are taken into account when discussing subtler neurofunctional correlates.

## Etiology

In most cases, AA follows ischemic or hemorrhagic strokes. Less frequently, it is associated with head trauma, encephalitis, tumor, or other causes [1].

In recent years, new facts have emerged on the clinical causes of AA, due to growing interest in some neurological conditions. The disorder has been reported in neurodegenerative disorders, such as Alzheimer’s disease [2–4] and frontotemporal dementia [5, 6]. In these cases, AA remains prominent until additional linguistic/cognitive deficits develop. AA has been reported in MELAS (Mitochondrial Encephalopathy, Lactic Acidosis and Stroke-like episodes) [7], a very rare, maternally inherited, genomic mitochondrial tRNA disorder due in most cases to a single base pair mutation (m.3243A>G). Brain and muscle tissue are most frequently affected, due to their high metabolic activity. In this condition, even minor physical efforts may precipitate repeated stroke-like events, ultimately leading to cognitive deterioration. Considering the extreme rarity of the disorder, AA is relatively frequent in MELAS e.g., [8–11]. AA is common also in the Kleffner-Landau Syndrome (KLS) [12], an ill-understood neurodevelopmental disorder typically appearing in children aged 3–8, possibly related to genetic factors such as GRIN2A mutations [13]. KLS is characterized by epilepsy and progressive loss of auditory language processing that may extend to other language skills including speech and sometimes responds to corticosteroid therapy.

## Lesion Lateralization in Pure Auditory Agnosias

If the 116 cases of AA retained for the present review are divided by clinical type of processing disorder, 63 patients (54.3%) presented with gAA, 39 (33.6%) with vAA, and 14 (12.1%) with nvAA. Damage affected cortical structures in 86 cases (74.1%) and was entirely subcortical in 30 (25.9%). In 4 subcortical cases, damage was restricted to the brainstem [14–17]. The hemispheric lesion profile in gAA, vAA, and nvAA is shown in Table 1. gAA, affecting both verbal and nonverbal auditory input processing, followed bilateral damage in 53/63 cases (84.1%), left hemispheric damage in 6 (9.5%), and right hemispheric damage in 4 (6.3%)^2^. vAA, selectively disrupting verbal input, was observed in 39 cases. Of these, 26 (66.7%) had bilateral lesions, 11 presented with left-sided damage (28.2%), and 2 were crossed aphasics with a right hemisphere lesion (5.1%). nvAA, selectively disrupting nonverbal input, was reported in only 14 cases. Damage was bilateral in 1 case (7.1%), left-sided in 6 (42.9%), and right-sided in 7 (50%). Overall, then, gAA frequently followed bilateral lesions, and vAA correlated strongly with bilateral or unilateral damage involving the language-dominant hemisphere. Lateralization is unclear in nvAA, which occurred in only one bilateral case, and in a similar number of left and right unilateral lesions.

**Table 1:** Lateralization of damage in generalized auditory agnosia (gAA), and in auditory agnosia selective for verbal (vAA) and nonverbal (nvAA) input processing

	Bilateral	Left hemisphere	Right hemisphere	Total
Generalized AA	53	6	4	63
Verbal AA	26	11	2*	39
Nonverbal AA	1	6	7	14
TOTAL	80	23	13	116

*Both patients were crossed aphasics

If observations are grouped by type of auditory input damage (verbal vs. nonverbal), different degrees of lateralization are observed (Table 2). Collapsing across gAA and vAA cases, verbal input processing disorders correlate very strongly with lesions in the language-dominant hemisphere, alone or in combination with similar contralateral lesions. This was true in 98/102 (96.1%) patients (59/63 with gAA, or 93.7% (the 4 outliers are the subjects in Footnote 2) and 39/39 with vAA, or 100%). Disorders followed cortical damage in 45/102 cases (44.1%), cortical-subcortical damage in 29/102 (28.4%), subcortical and brainstem damage in 18/102 (17.7%), and brainstem damage in 4/102 (3.9%). Nonverbal auditory input processing impairments are right-lateralized, but less strongly than verbal processing disorders are to left-sided damage. Collapsing across gAA and nvAA, difficulty with environmental sounds was associated with right hemisphere damage in 65/77 subjects (84.4%), resulting from 57/63 cases with gAA (90.5%) and 8/14 with nvAA (57.1%). Nonverbal processing deficits followed cortical damage in 30 cases (39%), subcortical damage in 25 (32.5%), and subcortical and brainstem damage in 12 (15.6%). No cases with damage restricted to the brainstem are on record.

**Table 2:** Number of patients with damage to the left and to the right hemisphere in the presence of impaired verbal (gAA + vAA) and nonverbal (gAA + nvAA) input processing

	Left hemisphere	Right hemisphere	Total
Verbal processing impairment	98	4*	102
Nonverbal processing impairment	12	65	77

*See Footnote 2

While the association between lesions in the left hemisphere (rather, in the language-dominant hemisphere) and disorders of speech sound processing is unquestionable, the correlation between right hemisphere damage and AA for nonverbal input is less stringent. nvAA has been described only in 14 cases. Eight of these are discussed in a single paper, which reports on nvAA following unilateral subcortical damage to the left (*n* = 4) and to the right hemisphere (*n* = 4) [18•]. Unfortunately, verbal input processing was not investigated in detail in these and in many other nvAA cases. Hence, results allow concluding that nvAA can result from damage to either hemisphere, but do not permit inferences on the relative pre-eminence of the right hemisphere in processing nonverbal stimuli—participants, and especially those with left-sided damage, may have also suffered from undetected verbal auditory processing deficits. The only clear instance of nvAA following left hemisphere damage was documented 10 years post-onset of a left parieto-temporal stroke in a patient with recovered gAA. In this case, extensive right-hemisphere BOLD activations during verbal tasks were consistent with improvement-related recruitment of premorbidly non-language brain regions [19•].

Taking available observations at face value, then, nvAA is associated to right hemisphere damage. However, evidence to this effect is unsatisfactory, for several reasons. Large right-hemisphere lesions extending to superficial and deep parietal and/or frontal structures frequently cause major motor and visuoperceptual deficits that attract greater attention than difficulties processing environmental sounds. Conversely, small right hemisphere lesions may yield only subtle auditory processing impairments that do not induce the patient to seek professional help. This latter possibility is supported by the observation that in several bilateral cases of AA a right hemisphere insult went unnoticed or yielded only a fleeting “hearing loss,” to be followed by full-fledged AA as the consequence of a second, left hemisphere stroke e.g., [20, 21]. Furthermore, while language skills are assessed by default in brain-damaged individuals, diagnosing nonverbal processing disorders requires specific, less commonly adopted testing procedures. For example, only a dedicated evaluation allowed detecting affective agnosia in patients with right hemispheric damage, whose verbal processing was intact [22, 23]. Methodological issues might play an important role, as even a cursory scrutiny of the literature on pure AA shows that verbal and nonverbal skills were frequently assessed with different accuracy. While language difficulties were evaluated in sufficient detail as to warrant the diagnosis of damaged speech input processing, nonverbal auditory processing skills were examined via simple clinical tasks, typically aimed at assessing the participant’s reaction to a limited number of familiar sounds but see [24••, 25••]. And, even when more formal evaluations were conducted, details on task structure and error analysis were frequently omitted. This could have led to misdiagnose gAA as vAA or to miss altogether mild forms of nvAA.

## Auditory Agnosia and the Temporal Lobes

In AA cases with cortical damage, one or both temporal lobes are affected (Table 1). Regardless of clinical type, cortical damage involves the primary and non-primary auditory cortices and subcortical lesions typically affect the acoustic radiations at the level of the putamen or deep to the insula; the lateral colliculi or the medial geniculate are involved less frequently [1].

In bilateral lesions, the account of pure AA is straightforward—left and right cortical and/or subcortical structures are damaged to such an extent that auditory stimuli can no longer be processed adequately.

The account of unilateral cases is more challenging. Hypotheses have traditionally focused on vAA. The disorder has been considered as a disconnection syndrome, in which left-hemisphere language areas are anatomically disconnected from both the left and the right auditory cortices, because of damage to intrahemispheric pathways and to transcallosal fibers, respectively [26•]. A reversal of the physiological right-ear advantage in dichotic listening tasks has been taken to support the disconnection hypothesis [24••, 27]. More recently, MRI and tractography studies have shown that damage to primary and nonprimary auditory cortices and to intrahemispheric and interhemispheric white matter pathways can be only partial, suggesting that functional, rather than anatomical disconnection may cause the disorder, perhaps due to asymmetric or asynchronous processing of verbal input in residual neural auditory structures e.g., [25••, 28, 29••].

Within the disconnection framework, accounting for pure nvAA in unilateral lesions is more problematic. By analogy with the account provided for vAA, right temporal lobe damage would have to prevent nonverbal input from reaching a (yet unknown) cortical center specialized for nonverbal sound recognition. And in left temporal lesions, damage should sever the connections to a cortical area involved in nonverbal sound processing, while sparing those linking the same area to the language network.

Further reports add to the uncertainty surrounding a thorough account of the physiopathological mechanisms underlying the reported AA cases. For example, in a small number of cases, damage was limited to the brainstem. In most of these, damage to acoustic pathways resulted in gAA [14–17, 30–32], but in 3 cases, pure vAA was observed [14, 16, 17]. While it is not surprising that brainstem lesions affect low-level, central auditory input processing across stimulus types, a disorder limited to verbal stimuli is unexpected.

To sum up, when scrutiny of the literature focuses on clinical distinctions, AA correlates to temporal damage and impaired verbal and nonverbal input processing to lesions of left and right temporal structures, respectively. However, there are enough unexpected or questionable observations as to warrant further investigations, especially on disorders of nonverbal processing.

## The Sources of the Auditory Input Processing Disorder

Two main hypotheses have been proposed to account for the observed dissociations of verbal vs. nonverbal sound processing and for their correlations with the left and the right temporal lobe, respectively. Both assume the disruption of lateralized processing mechanisms, distributed on a left/right continuum rather than in all-or-none fashion. On one view, the left hemisphere is more efficient at processing temporal features and the right hemisphere at processing spectral properties [33]. On the other, the left hemisphere processes sounds characterized by changes over very short time windows, and the right hemisphere sounds whose properties change more slowly [34]. On both accounts, verbal input has privileged access to the left hemisphere, either by virtue of intrinsic properties of the primary auditory cortex, or due to a different network-level organization of left and right auditory cortices [35].

Both views account for the correlation between vAA and left hemisphere damage, as speech sounds are characterized by fast changes in the soundwave, that are processed more efficiently in the left temporal cortex. They also accommodate the damage profiles documented in some vAA patients, in whom stops were affected more than longer consonants, and vowels were spared [20, 27]—stops are the shortest phonemes, and vowels the longest. On the other hand, selective difficulties with nonverbal sounds are less easily accommodated within either framework. For example, if selective nvAA stems from a difficulty processing slow-changing soundwaves or specific spectral sound features, left hemisphere skills should still permit processing nonspeech input, even in the presence of right temporal damage. However, this did not happen in patients with unilateral nvAA (see “Lesion Lateralization in Pure Auditory Agnosias”). Future studies will have to reconcile these puzzling issues also with fMRI studies suggesting that in neurotypical participants the primary auditory cortex responds to spectrotemporal modulations, but also that partially distinct portions of the superior temporal lobe are involved in processing different sound inputs [36–38], and that the superior temporal gyrus and sulcus are more responsive to auditory stimulus dimensions characterizing diverse auditory objects [39, 40].

The view that AA is caused by loss of spectrotemporal analysis, discussed in the previous paragraphs, would apply independent of whether the auditory cortices process sound features serially or in parallel. For many years, the prevailing view has been that sound input is processed sequentially—from simpler to more complex sound features and from the brainstem to the primary auditory cortex to nonprimary cortices and associative cortical areas [41, 42, 43•, 44–46]. In contrast, recent studies based on direct cortical stimulation in candidates for awake brain surgery suggest parallel processing and question the roles traditionally assigned to primary and nonprimary cortices [47••]. A similar tension exists in the functional domain between the models that assume sequential processing of auditory inputs and those that hypothesize a quasi-continuous auditory processing from acoustic input to semantic auditory representations [43•, 48•].

## The Locus of the Lateralization of Auditory Input Processing

AA correlates with lateralized hemispheric damage—verbal auditory processing deficits are strongly linked to left-sided lesions and nonverbal deficits are likely to result from right-sided lesions. However, auditory agnosias have been reported following damage to any components of the auditory pathway, from the brainstem to the auditory cortex. While it has been assumed that the critical locus of damage corresponds to the auditory cortices, pinpointing the neural structures whose damage is primarily or directly responsible for lateralized deficits is difficult. Taking issue with speech processing and vAA may prove useful.

It is reasonable to assume that low-level properties of the speech input (basic spectral and temporal patterns, location in space, intensity, etc.) are processed symmetrically in the brainstem, independent of their nature,^3^ and that at a later stage, when complex feature bundles corresponding to more abstract sound representations are integrated, acoustic input is treated as speech (as opposed to animal voice, music, environmental noise, and so on) and processed in the left hemisphere. On this view, since the vAA cases considered in the present review (Tables 1 and 2) consistently suffer from lesions in the primary auditory cortex of the language-dominant hemisphere, it would be tempting to conclude that this is the neural site responsible for selective disorders of speech input processing. Yet, the role of damage to this region in unilateral vAA can still be questioned on two accounts. First, the Heschl’s gyrus and its outgoing connections are partly spared in several cases with cortical damage e.g., [29••, 49, 50]. Second, lesions in most cases involve also the nonprimary auditory cortex and extend to other structures, like the posterior portions of the middle temporal gyrus, the supramarginal gyrus, and the angular gyrus, that are part of the language network see [24••, 25••, 28, 51•]. These facts invite considering the possibility that auditory input is processed symmetrically up to the primary (and nonprimary?) auditory cortex, and that only damage to associative language areas yields lateralized speech input disorders—i.e., vAA. Needless to say, an analogous reasoning applies to disorders of nonverbal auditory processing that follow right hemisphere lesions.

## Factors That Further Complicate the Interpretation of AA

Not only are the neural and functional disorders directly responsible for AA heterogeneous—co-occurring difficulties may further complicate the clinical pictures and their interpretation.

A first factor, whose influence is still largely ignored, concerns the potential role of the corticofugal auditory pathways (for reviews, see [52, 53•]). Throughout the auditory system, connections between centripetal and centrifugal pathways set up neurofunctional loops that interact pervasively at all relay stations (primary and nonprimary auditory cortex, inferior colliculi, medial geniculate, cochlear nucleus, and olivary system). The top-down influence of the corticofugal system constrains activity even at peripheral stages. For example, activation of the cortico-olivocochlear pathway can modulate cochlear responses and affect speech processing [53•, 54]. The ways and the extent in which this influence is exerted under physiological and pathological conditions must be considered carefully in individuals with AA, especially because descending and ascending pathways are adjacent, and therefore likely to be damaged simultaneously with hard-to-define consequences. With regard to this issue, of particular interest are the auditory dysfunctions of unclear origin reported on in some stroke-related AA (e.g., hyperacusis, misophonia, and language echo, e.g., [55–57]; for reviews, see [58, 59]).

Peripheral hearing disorders may also complicate the interpretation of AA. Albeit present in many AA cases, neurosensory hearing loss is usually so mild as to be reasonably deemed irrelevant. However, significant hearing loss is common in rare conditions associated with AA like MELAS [8–11]) and Landau-Kleffner Syndrome [12] and has been documented in some AA cases [6, 60•, 61–67]. At issue here is whether these disorders are a mere co-occurrence in AA or rather constrain/modify significantly its manifestations.

Attentional (and perhaps also other cognitive) impairments could also influence the clinical manifestations of AA by modulating if not the nature, at least the severity of the disorder. Consider for example cortical deafness, in which the patient does not respond to auditory stimuli or does so haphazardly. Cortical deafness can be the presenting sign of an auditory processing disorder, but often evolves toward less severe forms (gAA, vAA, and nvAA) [57, 68, 69]. Intriguingly, in several bilateral cases with persistent cortical deafness, right hemisphere damage involved structures critical for attentional mechanisms [28, 70–72], and in a recent case, anosognosia has been reported in a patient with severe AA [73]. In addition, right-sided damage to temporal, but also parietal and frontal regions, can cause auditory neglect [74] whose presence may enhance auditory processing difficulties. These reports invite considering the possibility that attentional deficits caused by damage to primarily non-auditory areas of the right hemisphere play a significant role in the clinical manifestations of auditory agnosia, at least in bilateral cases.

## Auditory Agnosias Are Neurally and Functionally Heterogeneous

If it is accepted that AA can result from damage to any relay station along the auditory pathways, and that each relay station processes different (sets of) properties of the auditory input, it must also be acknowledged that AA cannot be a unitary phenomenon.

The reports considered for the present review indicate that AA can be caused by damage to different levels of the auditory pathways, and that even lesions affecting brainstem, collicular, or genicular structures, where processing is presumably non-lateralized, may result in AA and even in selective forms of the disorder. Under these circumstances, disruption of low-level properties processed similarly in the two halves of the auditory system could carry over to higher stations of the auditory pathway and, if low-level processing damage affects sound properties typical of specific classes of auditory objects, emerge as a selective form of AA. This could account for the surprising instances of vAA or nvAA following brainstem damage. Of course, the same behavioral profile of auditory processing damage could be caused by lesions of cortical structures engaged in high-level auditory processing—i.e., levels where speech sounds are treated as phonemes, and nonverbal stimuli as music.

The heterogeneity of AA is obvious not only at the neural level but also at the behavioral level, both quantitatively and qualitatively. Disorders of auditory input processing range in severity from complete cortical deafness to selective impairments affecting different features of the auditory input. Pure vAA can result from damage to prephonemic or phonemic properties of speech sounds [20, 24••, 51•, 75, 76] or from the inability to process fast as opposed to slow-changing stimuli [54, 56]. Similarly, selective nonverbal auditory processing deficits have been observed not only for environmental sounds [19•] but also for emotional speech [23, 77] and music (see, e.g., [78] and for review [79]). In short, any neurofunctional account of AA must accommodate a large range of neural lesions and behavioral disorders. Forthcoming studies of individuals with pure AA should provide detailed reconstructions of brain damage and thorough investigations of auditory input processing, from basic perception to the recognition of abstract entities. Fine-grained assessments of this type will help associate specific cortical and subcortical structures with the processing of specific sound features.

## Conclusions

Pure AA is a very rare condition, in which damage to central auditory pathways selectively disrupts auditory input processing. The disorder may selectively affect verbal sounds (vAA), nonverbal sounds (nvAA), or both (gAA). Despite the relatively straightforward characterization of the disorder and of its clinical varieties, many critical issues are still open. Since AA is neurally heterogeneous, forthcoming investigations will have to accurately characterize brain damage in each case. Since the disorder is also functionally heterogeneous and clinical phenotypes are distributed along a quantitative and qualitative continuum, behavioral single-case investigations will have to be equally exhaustive. Studies will have to carefully reconstruct neural damage, establish on a case-by-case basis if AA results from damage to basic properties of the sound input or to more abstract sound representations, identify the stimulus properties affected, and verify if the processing of verbal and nonverbal stimuli is disrupted to the same or a different degree. Thorough neural and functional analyses are the prerequisites for the development of hypotheses correlating damage to specific components of the auditory pathway and disorders of specific stages of sound processing. They will permit, among other things, to establish the critical locus responsible for sound category-specific lateralized effects. The clinical and neural characteristics of selective nonverbal processing disorders, as well as their clinical status, will deserve particular attention given the relative indeterminacy of available data.

Future studies will also have to identify in each case the “core” AA traits, by considering the potential interference of neurosensory hearing loss and of additional cognitive disorders on the clinical manifestations of AA. The influence of the corticofugal auditory pathways will have to be considered.

Progress in this area will continue to be slow, owing to the rarity of the disorder, that rules out group studies as a viable approach to answer outstanding questions. Notwithstanding these difficulties, the possibility to thoughtfully combine audiological, neuropsychological, and neuroimaging data in single brain-damaged individuals with evidence from neurotypical subjects (e.g., neuroimaging, electrophysiologic, and psychoacoustic investigations) affords unprecedented opportunities for progress in the field.

### Supplementary Information

ESM 1 (DOCX 64.5 kb)

## Data Availability

All the papers used for the present review are listed in the Supplementary Material.

## Abbreviations

AA: auditory agnosia (difficulty processing auditory input in general)
gAA: generalized auditory agnosia (difficulty processing all types of acoustic input)
vAA: verbal auditory agnosia (difficulty processing verbal auditory input – aka word deafness)
nvAA: nonverbal auditory agnosia (difficulty processing nonverbal auditory input – aka environmental sound agnosia)

## Footnotes

1 No further distinctions are made in the present review among types of environmental sounds (e.g., between human and animal vocalizations, action sounds, object sounds, and music).

2 In these four patients, the damaged right hemisphere may have been involved in language processes premorbidly. One was a left hander [80]. In another, whose handedness was unspecified, misophonia had been diagnosed in adolescence [81]. A right-hander with corticobasal degeneration (usually associated with bilateral atrophy) showed right-sided neuroradiological evidence on MRI, PET, and DAT-scan, but also produced phonemic paraphasias [82]. Finally, a right-hander presented paraphasic speech and naming difficulties following a right-hemisphere stroke [55].

3 An additional reason to assume substantially symmetrical processing at least up to the medial geniculate bodies is that from the medulla (superior olivary complex, dorsal and ventral cochlear nuclei) to the brainstem (inferior colliculi) fibers of the auditory pathways project both homolaterally and contralaterally.
