# Supplementary material for: The Auditory Agnosias: a Short Review of Neurofunctional Evidence
Source: Curr Neurol Neurosci Rep. 2023 Sep 25;23(11):671–9. doi: 10.1007/s11910-023-01302-1 (PMC10673750; doi:10.1007/s11910-023-01302-1)
Supplement: Supplementary file 1 — ESM 1 (DOCX 64.5 kb) [file 11910_2023_1302_MOESM1_ESM.docx]

The list of the 116 studies published between 1893 and December 2022. For the studies quoted in the text, the reference number is reported in square brackets. The sequence of the lesions is indicated in bilateral cases (L>R = left, than right lesion; R>L = right, then left lesion).

| **Paper information** | | **Patient information** | | | **Type of auditory Agnosia** | **Anatomical Data** | | | | | |
| --- | --- | --- | --- | --- | --- | --- | --- | --- | --- | --- | --- |
| *Authors*  *(publication year)* | *Journal* | *Age* | *Sex* | *Handedness* |  | *Etiology* | *Number of Lesions* | *Hemisphere damaged*  *(injury report)* | *Brain structures involved* | | |
|  |  |  |  |  |  |  |  |  |  | *Left Hemisphere* | *Right Hemisphere* |
| Asakuno & Ishida (2014) [53] | Surg Neurol Int | 55 | F | R | gAA | CVA | 1 | (R>L) | Cortical | STG | insula |
| Auerbach et al (1982) [58] | Brain | 58 | M | R | gAA | CVA | 2 | LR (R>L) | Cortical-subcortical | T + T isthmus + WM | T-P + T isthmus + WM |
| Barret (1910) | J Nerv Ment Dis | 67 | M | R | gAA | CVA | 1 | Bil | Cortical-subcortical | T + subcortical | T + subcortical |
| Bervini et al (2014) [16] | Clin neurol Neurosurg | 31 | F | R | vAA | VOGA resection | 1 | Bil | Brainstem | Inferior colliculi | Inferior colliculi |
| Best & Howard (1994) | Aphasiology | 51 | F | R | gAA | CVA | 2 | LR | Cortical | T-P | T-P |
| Bhaskaran et al (1998) | J Assoc Ohysicians India | 53 | M | R | vAA | CVA | 1 | R | Cortical | - | T-P + F |
| Brick et al (1985) | Neurology | 49 | M | - | vAA | CVA | 2 | LR (R>L) | Subcortical | Claustrum, external capsule, putamen, AR and WM of STG | Claustrum, external capsule, putamen, AR and WM of STG |
| Buchman et al (1986) [25] | J Neurol Neurosurg Psychiatry | 75 | F | R | vAA | CVA + AD | 2 | RL (R>L) | Cortical-subcortical | P + T + T isthmus + WM + putamen +  WM of O | P + WM + T + extreme capsule, claustrum, external capsule, internal capsule, visual and AR |
| Buchtel & Stewart (1989) | Brain Lang | 51 | M | R | gAA | CVA | 2 | LR | Cortical | F-T | T |
| Clarke et al (2000) | Neuropsychologia | 54 | M | R | gAA | CVA | 1 | L | Cortical | T | - |
| Clarke et al (2000) | Neuropsychologia | 62 | F | R | nvAA | CVA | 1 | L | Cortical | T-F | - |
| Clarke et al (2000) | Neuropsychologia | 64 | F | R | gAA | CVA | 1 | L | Cortical | T-F-P + insula | - |
| Coslett et al (1984) | Neurology | 48 | M | R | vAA | CVA | 2 | RL(R>L) | Cortical | T | T-P-F |
| Dalla Pria et al (1979) | Schweiz Arch Neurol Neurochir Psychiatr | 40 | F | - | vAA | CVA | 2 | RL | Cortical | T-P | T |
| Déjérine & Sérieux (1987) | Revenue de Psychiatrie | 43 | F | R | vAA | FTD | - | Bil | Cortical-subcortical | T + WM | T + WM |
| Earnest et al (1977) | Neurology | 27 | M | L | gAA | CVA | 2 | RL | Cortical | T-P | T-P |
| Engelien et al (1995) [81] | Brain | 52 | M | R | gAA | CVA | 2 | LR (L>R) | Cortical-subcortical | F + P + T + insula + basal ganglia + WM | T + insula |
| Eustache et al (1990) | Neuropsychologia | 31 | M | R | gAA | CVA | 1 | L | Cortical | P+T | - |
| Eustache et al (1990) | Neuropsychologia | 64 | M | R | nvAA | CVA | 1 | R | Cortical-subcortical | - | Lenticular nucleus, external capsule + F + diffuse atrophy |
| Fletcher et al (2013) | Neuropsychologia | 67 | F | R | gAA | PPA | - | Bil (L>R) | Cortical | Peri-Sylvian atrophy |  |
| Fletcher et al (2013) | Neuropsychologia | 71 | F | R | gAA | PPA | - | Bil (R>L) | Cortical | Anterior temporal atrophy | Anterior temporal atrophy |
| Fujii et al (1990) | Cortex | 55 | M | R | nvAA | CVA | 1 | R | Cortical | - | T |
| Garde & Cowey (2000) | Cortex | 26 | F | R | gAA | CPM, EPM | - | Bil | Cortical - Brainstem | Pons + T | Pons + T |
| Godefroy et al (1995) [71] | Cortex | 58 | F | R | gAA | CVA | 2 | Bil | Subcortical | External capsule + T isthmus | External capsule + T isthmus |
| Greve et al (2004) | J Neurol Neurosurg Psychiatry | 45 | M | - | vAA | CVA | 2 | RL | Cortical | T | T |
| Gutschalk et al (2015) | Cortex | 51 | M | R | gAA | CVA | 3 | RL (R>L) | Cortical-subcortical | T | T + WM of planum T + P |
| Habib et al (1995) [32] | Neuropsychologia | 44 | F | R | nvAA | CVA | 2 | RL | Cortical-subcortical | Insula + WM | T + insula |
| Hattiangadi et al (2005) | Brain Lang | 12 | M | - | gAA | HT | 1 | Bil | Cortical-subcortical | T + WM of F+ P + O + splenium + thalamus + caudate nucleus | T + WM of F + internal capsule + caudate nucleus + right thalamus |
| Hayashi & Hayashi (2007) | Clin Neurophysiol | 59 | M | R | vAA | CVA | 2 | L | Subcortical | Thalamus + putamen + AR | - |
| Hayashi & Hayashi (2007) | Clin Neurophysiol | 59 | M | R | vAA | CVA | 2 | RL (L>R) | Subcortical | Putamen + internal capsule + T isthmus | Putamen |
| Holmes et al (2021) [19] | Cortex | 33 | F | - | gAA | CVA | 1 | R | Cortical-subcortical | - | P + T + insula |
| Iizuka et al (2007) | Eur J Neurol | 66 | M | R | vAA | PPA | - | Bil | Cortical | Perisylvian atrophy |  |
| Ishii et al (1995) | Inter Med | 55 | M | R | gAA | CVA | 2 | LR | Cortical-subcortical | T | T-P |
| Jeon et al (2021) [72] | Korean J Otorhinolaryngol-Head Neck Surg | 72 | F | R | vAA | CVA | 2 | RL | Cortical | P-T | T |
| Johkura et al (1998) [15] | J Neurol Sci | 46 | M | R | gAA | HT | 1 | L | Cortical- Brainstem | F + inferior colliculi | - |
| Jörgens et al (2008) | Neurocase | 71 | R | R | vAA | Cerebral degeneration | - | Bil | Cortical | Generalized atrophy | Generalized atrophy |
| Joswig et al (2015) [17] | Clin Neurol Neurosurg | 19 | M | - | vAA | Post tumor surgery | - | Bil | Brainstem | Inferior colliculi | Inferior colliculi |
| Kaga et al (2005) | Acta Otolaryngol | 43 | M | - | gAA | CVA | 2 | RL | Subcortical | AR | AR |
| Kaga et al (2000) | Acta Otolaryngol | 37 | M | - | gAA | CVA | 2 | RL | Cortical-subcortical | T + medial geniculate body + Broca’s area + P + insula + external capsule | T + medial geniculate body |
| Kanshepolsky et al (1973) | Neurology | 62 | M | R | gAA | CVA | 3 | LR | Cortical-subcortical | T + basal ganglia + internal capsule | T |
| Kawasaki et al (2013) | Acta Med Nagasaki | 65 | M | R | gAA | CVA | 1 | R | Cortical | Sylvian fissure | T + F |
| Kazui et al (1990) | Brain Lang | 66 | M | R | gAA | CVA | 2 | LR | Cortical | P | T + insula |
| Kim et al (2011) [2] | J Clin Neurol | 59 | F | R | vAA | AD | - | Bil (L>R) | Cortical | T | T |
| Klarendic et al (2021) [83] | Cortex | 66 | F | R | gAA | CVA | 2 | RL | Cortical-subcortical | T + WM | F + insula |
| Kneebone & Burns (1981) | Clin Exp Neurol | 79 | M | R | gAA | CVA | 2 | RL(R>L) | Cortical | T-P | T-P |
| Koyama & Domen (2016) | Prog Rehabil Med | 59 | F | R | gAA | CVA | 2 | LR | Subcortical | AR | Putamen + thalamus + AR |
| Leng et al (2013) [9] | Mitochondrial DNA | 14 | M | - | vAA | MELAS | - | Bil | Cortical-subcortical- Brainstem | Basal ganglia + midbrain + T | Basal ganglia + midbrain + T |
| Lin et al (2022) [11] | Front Neurol | 43 | M | R | vAA | MELAS | - | Bil | Cortical | T | T |
| Mackenzie et al (1980) | Aust J Human Commun Disord | - | F | R | gAA | CVA | 2 | LR | Cortical | insula | P-T |
| Maffei et al (2017) [33] | Cortex | 38 | F | R | vAA | CVA | 1 | L | Cortical-subcortical | T + insula + P + AR + temporal longitudinal fasciculus + temporal connections of corpus callosum | - |
| Maneta et al (2001) | Int J Lang Commun Disord | 79 | M | - | vAA | CVA | 1 | L | Cortical | P | - |
| Mark & Chobor (1990) | Aphasiology | 43 | M | R | gAA | CVA | - | (R>L) | Cortical-subcortical | T + WM + hippocampus + thalamus + P | T + WM + insula + claustrum +(Thalamus) + P |
| Marshall et al (1985) | Brain Lang | 62 | F | R | gAA | CVA | - | (L>R) | Cortical-subcortical | T + AR + P | T + AR |
| Mashima et al (2021) [20] | Neurol Sci | 73 | F | R | gAA | CBD | - | R | Cortical-subcortical | - | T + striatum |
| Masuda et al (2000) | Ann Otol Rhinol Laryngol | 48 | F | R | vAA | Tumor Surgery | - | Bil (L>R) | Subcortical - Brainstem | Inferior colliculi + medial geniculate body | Inferior colliculi + medial geniculate body |
| Mendez & Rosenberg (1991) | J Am Geriatr Soc | 65 | F | - | vAA | CVA | 1 | L | Cortical | T | - |
| Mendez & Geehan (1988) [78] | J Neurol Neurosurg Psychiatry | 60 | M | R | gAA | CVA | 2 | RL | Cortical | T | T |
| Mendez & Geehan (1988) [78] | J Neurol Neurosurg Psychiatry | 23 | M | RL | gAA | CVA | 2 | RL | Cortical | P-T | F-P-T |
| Mendez (2001) [18] | Cortex | 68 | M | L | gAA | CVA | 1 | R | Cortical | - | T-P |
| Metz-Lutz & Dahl (1984) | Brain Lang | 24 | F | R | vAA | CVA | 1 | L | Cortical-subcortical | T | - |
| Meyer et al (1996) [14] | J Neurol Neurosurg Psychiatry | 36 | M | - | vAA | Post tumor surgery | - | Bil | Brainstem | Inferior colliculi | Inferior colliculi |
| Miceli et al (2008) [8] | Neurol Sci | 38 | F | R | gAA | MELAS | - | Bil | Cortical | T | T |
| Miceli (1982) [24] | Neuropsychologia | 53 | F | R | gAA | CVA | 2 | RL (R>L) | Cortical | T-O | T |
| Michel et al (1980) | Brain Lang | 40 | M | R | gAA | CVA | 2 | ? | Cortical | T | T-P |
| Motomura et al (1986) | Brain | 69 | M | R | gAA | CVA | 2 | LR | Subcortical | Thalamus | Thalamus + Internal capsule |
| Musiek et al (2004) | Int J Audiol | 21 | - | - | gAA | Neisseira meningitidis + CVA | 1 | L | Cortical- Brainstem | Cerebellum-O + T + Inferior colliculi | Inferior colliculi |
| Nakakoshi et al (2001) | Brain Lang | 42 | M | R | vAA | CVA | 1 | L | Subcortical | Putamen | - |
| Nedelec-Ciceri et al (1996) | Rev Neurol | 80 | F | R | vAA | CVA | 1 | R | Cortical | - | T-P |
| Oppenheimer & Newcombe (1978) | Arch Neurol | 65 | M | R | gAA | CVA | 3 | LLR | Cortical | F-P-T | T-P |
| Otsuki et al (1998) | Eur Neurol | 67 | M | R | gAA | Cerebral degeneration | - | Bil | Cortical | Generalized atrophic changes  (> T) | Generalized atrophic changes |
| Palma et al (2012) [54] | J Neurol | 52 | M | R | vAA | Paraneoplastic encephalitis | - | L | Cortical | T + insula | - |
| Pan et al (2004) [34] | Neurology | 14 | M | - | gAA | N | - | Bil | Cortical-Subcortical-Brainstem | Inferior colliculi | Inferior colliculi + thalamus + F |
| Papathanasiou et al (1998) [66] | In J Lang Commun Disord | 75 | M | R | vAA | CVA? | - | L | Cortical | P | - |
| Parving et al (1980) | Scand Audiol | 76 | M | R | gAA | CVA | ? | Bil | Cortical | T | T |
| Pinard et al (2002) [27] | Neurocase | 38 | F | - | gAA | PPA | - | Bil | Cortical-subcortical | Atrophy + WM of cerebellum | WM of cerebellum |
| Poliva et al (2015) [36] | Cogn Behav Neurol | 28 | F | - | gAA | HT | - | Bil | Subcortical-Brainstem | inferior colliculi + red nucleus medial geniculate body | Basal ganglia + putamen + pons |
| Praamastra et al (1991) [59] | Brain | 57 | M | R | gAA | CVA | 2 | LR (L>R) | Cortical | T-F-P | T |
| Roberts et al (1987) [21] | J Neurol Neurosurg Psychiatry | 61 | M | R | gAA | CVA | 1 | R | Cortical | - | T-P |
| Robson et al (2012) | Aphasiology | 73 | F | R | gAA | CVA | 2 | RL (R>L) | Cortical | P-O | O-P-T |
| Rosati et al (1982) | J Neurol | 49 | M | R | gAA | CVA | 2 | LR (L>R) | Cortical | T | T |
| Salemme et al (2022) [4] | Neurol Sci | ? | M | R | gAA | AD | - | Bil (R>L) | Cortical | T-P atrophy | T-P atrophy |
| Saygin et al (2010) [23] | Neuropsychologia | 74 | M | R | nvAA | CVA | 1 | L | Cortical | T-P | - |
| Schuster & Hans Taterka (1926) | Z Gesamte Neurol Psychiatr | 68 | F | - | gAA | CVA | ? | (L>R) | Cortical-subcortical | F-P + WM of T + thalamus + putamen + internal capsule | WM + internal capsule |
| Shin et al (2004) | J Korean Neurol Assoc | 18 | F | - | vAA | MELAS | - | LR | Cortical-subcortical | T-P-O + basal ganglia | T-P-O + basal ganglia |
| Shivashankar et al (2001) | Clin Neurol Neurosurg | 50 | M | R | vAA | N | - | Bil | Subcortical | Medial geniculate body + internal capsule | Medial geniculate body + internal capsule |
| Shivashankar et al (2001) | Clin Neurol Neurosurg | 24 | M | R | vAA | N | - | Bil | Subcortical | Medial geniculate body + internal capsule | Medial geniculate body + internal capsule |
| Slevc et al (2011) [29] | Neuropsychologia | 66 | M | R | gAA | CVA | 1 | L | Cortical | T-P | - |
| Smith et al (2019) [10] | Neurologist | 61 | F | R | vAA | MELAS | - | Bil | Cortical | T | T |
| Spreen et al (1995) | Arch Neurol | 65 | M | R | nvAA | CVA + sarcoma | 1 | R | Cortical | - | F-T-P + insula |
| Stefanatos et al (2005) [28] | J Int Neuropsychol Soc | 43 | F | R | vAA | CVA | 1 | L | Cortical-subcortical | T + WM + insula + F-P | - |
| Sugiura & Torii (2017) | Case Rep | 55 | M | R | vAA | CVA | 2 | RL | Subcortical | Putamen | Putamen |
| Suh et al (2012) | Ann Rehabil Med | 73 | M | R | gAA | CVA | 2 | RL | Subcortical | Basal ganglia + internal capsule + WM of T (AR) | Basal ganglia |
| Sung et al (1997) |  | 36 | - | R | vAA | CVA | ? | Bil | Cortical | T | T |
| Tabuchi et al (2007) | J Neurosurg | 52 | F | R | gAA | CVA | 2 | (R>L) | Cortical-subcortical | T + P + O | Insula + T + AR |
| Tack et al (1986) | Acta Neurol Belg | 64 | M | R | gAA | CVA | 1 | L | Cortical | Peri-insular | - |
| Takahashi et al (1992) | Cortex | 55 | M | R | vAA | CVA | 1 | L | Subcortical | Thalamus + internal capsule + WM of T-P (AR) | - |
| Tanaka et al (2002) [22] | Cortex | 57 | M | R | nvAA | CVA | 1 | R | Subcortical | - | Putamen + WM + AR |
| Tanaka et al (2002) [22] | Cortex | 58 | M | R | nvAA | CVA | 1 | R | Subcortical | - | Putamen + WM + AR |
| Tanaka et al (2002) [22] | Cortex | 64 | M | R | nvAA | CVA | 1 | R | Subcortical | - | Putamen + WM + AR |
| Tanaka et al (2002) [22] | Cortex | 59 | F | R | nvAA | CVA | 1 | R | Subcortical | - | Putamen + WM + AR |
| Tanaka et al (2002) [22] | Cortex | 65 | F | R | nvAA | CVA | 1 | L | Subcortical | Putamen + WM + AR | - |
| Tanaka et al (2002) [22] | Cortex | 62 | M | R | nvAA | CVA | 1 | L | Subcortical | Putamen + WM + AR | - |
| Tanaka et al (2002) [22] | Cortex | 59 | F | R | nvAA | CVA | 1 | L | Subcortical | Putamen + WM + AR | - |
| Tanaka et al (2002) [22] | Cortex | 47 | M | R | nvAA | CVA | 1 | L | Subcortical | Putamen + WM + AR | - |
| Tanaka et al (1987) | Brain | 26 | F | R | gAA | CVA | 2 | LR (R>L)) | Cortical-Subcortical | T-P + AR | T-P + AR |
| Taniwaki et al (2000) | Clin Neurol Neurosurg | 46 | M | R | gAA | CVA | 2 | LR | Subcortical | Putamen + AR | Putamen + AR |
| Tessier et al (2007) [35] | Brain Inj | 65 | F | R | gAA | CVA | 2 | (R>L) | Subcortical- Brainstem | internal capsule + WM | Medulla + pons + mesencephalum + internal capsule + WM |
| Utianski et al (2018) [5] | Cortex | 65 | F | R | gAA | PPA (BD) | - | Bil (L>R) | Cortical | F-T-P | F-T-P |
| Verma & Post (2013) | JAMA Neurol | 29 | F | R | vAA | HIV | - | Bil | ? | Multiple lesions including T | Multiple lesions including T |
| von Stockert (1982) | Brain Lang | 23 | M | - | gAA | HT | - | Bil | Cortical | T-P | T |
| Walters et al (2022) | Can J Neurol Sci | 21 | F | - | gAA | Autoimmune Encephalitis | - | Bil | Cortical-Subcortical | Thalamus + caudate nucleus + T + F | Thalamus + caudate nucleus + T |
| Watanabe et al (2020) [6] | Front Neurol | 80 | - | R | gAA | PPA | - | Bil (L>R) | Cortical | T-F | T-F |
| Wohlfart et al (1952) | J Nerv Ment Dis | 50 | M | R | gAA | MS | - | Bil | Cortical-subcortical | O + F + T + insula | T + cerebellar + F + P |
| Wolmetz et al (2010) [55] | J Cogn Neurosci | 68 | M | RL | vAA | CVA | 1 | L | Cortical-subcortical | T + WM +P | - |
| Yaqub et al (1988) | Brain | 38 | M | R | vAA | CVA | 2 | (R>L) | Cortical-subcortical | T | T-P |
| Zhu et al (2010) | J Zhejiang Univ Sci | 19 | F | R | vAA | EPM | - | Bil | Subcortical | Putamen + caudate nucleus + splenium | Putamen + caudate nucleus + splenium |

**Patient information**: F = female; M = male; **Anatomical Data**: *Etiology*: AD = Alzheimer dementia; CBD = corticobasal degeneration; CPM = central pontine myelinolysis; CVA = cerebrovascular accident; EPM = extra pontine myelinolysis; FTD = frontotemporal dementia; HT = head trauma; HIV = Human Immunodeficiency Virus; MELAS = Mitochondrial Encephalopathy Lactic Acidosis Stroke; MS = Multiple Sclerosis; N = neoplasm; PPA = Primary Progressive Aphasia; VOGA = Vein of Galen aneurysm; *Hemisphere damaged:* Bil: bilateral; L = left; R = right; *Brain structures involved:* AR = auditory radiations; O = occipital structures; P = parietal structures; F = frontal structures; T = temporal structures; WM = white matter.
